# Methadone Concentrations in Exhaled Breath Condensate, Serum and Urine of Patients Under Maintenance Treatment

**Published:** 2017

**Authors:** Maryam Khoubnasabjafari, Khalil Ansarin, Vahid Jouyban-Gharamaleki, Vahid Panahi-Azar, Samin Hamidi, Zhila Azarmir, Abolghasem Jouyban

**Affiliations:** a *Tuberculosis and Lung Disease Research Center, Tabriz University of Medical Sciences, Tabriz 51664, Iran. *; b *Department of Mechatronic Engineering, International Campus, University of Tabriz, Tabriz 51664, Iran.*; c *Liver and Gastrointestinal Diseases Research Center, Tabriz University of Medical Sciences, Tabriz 51664, Iran. *; d *Drug Applied Research Center, Tabriz University of Medical Sciences, Tabriz 51664, Iran.*; e *Neurosciences Research Center, Tabriz University of Medical Sciences, Tabriz 51664, Iran.*; f *Pharmaceutical Analysis Research Center and Faculty of Pharmacy, Tabriz University of Medical Sciences, Tabriz 51664, Iran.*; g *Kimia Idea Pardaz Azarbayjan (KIPA) Science Based Company, Tabriz University of Medical Sciences, Tabriz 51664, Iran.*

**Keywords:** Methadone, Exhaled breath condensate, HPLC, Serum, Urine, Correlation

## Abstract

Drug abuse is a serious problem causing health, economical and psycho-social negative outcomes. Methadone is commonly used drug for management of drug addiction. Exhaled breath condensate (EBC) is a promising non-invasive biological sample which attracted more attention in recent years. This work aimed to extend the applicability of a developed preconcentration – liquid chromatographic method for analysis of methadone in serum and urine samples. Drug concentrations in EBC, serum and urine are also investigated for dose-concentration and their inter-correlations. Biological samples were collected from 53 patients receiving methadone and the concentrations were determined using a validated analytical method after a pre-concentration step. Methadone measured in all samples and there are correlations between administered dose of methadone and its serum and urine concentrations. A weak correlation is observed between dose and EBC concentration. Wider variations in EBC concentrations of methadone could be justified concerning a number of affecting parameters such as relative humidity of the collection area and further investigations are required for standardization of EBC as a non-invasive biological sample to be used in clinical practice.

## Introduction

Drug abuse is a serious problem causing health, economical and psycho-social negative outcomes. A well established therapeutic regime called methadone maintenance treatment (MMT) is the widely used method for management of drug addiction. Methadone has some properties which make it an effective therapeutic agent for treatment of abused drugs withdrawal syndrome; 1) methadone is an orally active compound, 2) possesses high bioavailability, and 3) long half-life (mean values of 28 h) which allows administration of a single dose every day and reaches to the steady state serum level after several administrations (1). It should be noted that the half-life may be further prolonged in opiate users (2). On the other hand, its pharmacokinetics is variable and there are considerable inter-individual variations of plasma concentrations following administration of the same dose (3, 4). Plasma or other biological sample concentrations of methadone differ possibly due to variations in genetic, physiological, pathological and pharmacological factors (5). Methadone’s oral bioavailability was found to be 84 ± 26%, elimination half life 30 ± 7.7 h, plasma clearance 96 ± 30.6 mL/min, protein binding 60-90 % and volume of distribution of 3.6 ± 0.7 L/Kg (6). It is metabolized by a subgroup of P450, i.e. CYP3A4, in liver and gastrointestinal mucosa and co-administration of other drugs may affect its metabolism.

Most of patients under MMT complain on their methadone dosage and higher doses of methadone are requested and a reliable and easily measurable parameter is demanded in clinical practice for methadone dose adjustment. On the other hand, despite of methadone’s proven efficacy in improving quality of life of drug abusers and reducing the morbidity and mortality associated with continued use of heroin and other illicit drugs (7), around 30% of opiate-related deaths in US is due to methadone poisoning where methadone is only prescribed in relative frequency of 5% (8). The current dose adjustment method in MMT centers is clinical observations and interview to search for withdrawal symptoms; intoxication, euphoria, sedation, shortness of breath etc. and measurement of plasma/serum concentrations of methadone (3, 9-14). Serum or plasma and urine samples are the most commonly used biological samples for monitoring methadone levels. Exhaled breath condensate (EBC) is another simple and non-invasive sampling method to be used for therapeutic drug monitoring (TDM) purposes. 

The EBC contained water, volatile compounds in gaseous phase and non-volatile compounds carried in the aerosol particles from the respiratory tract. EBC was found as the higher detection rate method for drug addiction when compared with self-report, blood and urine sampling procedures. It was also shown that EBC could be used for detecting the intake of the most commonly abused drugs (15). The results of a questionnaire analysis revealed that EBC is more popular than urine sampling and 87% of the patients preferred donating EBC over urine sample (16).

This work is aimed to extend the applicability of a recently developed analytical method for quantification of methadone in EBC (17) for determination of methadone in urine and serum samples and to investigate the reliability of serum, urine and EBC samples for monitoring their relationships with the administered dose of methadone. Cross correlations among the methadone concentrations in these biological samples are also studied.

## Experimental

*Chemicals*

Methadone hydrochloride was purchased from TEMAD Company (Tehran, Iran). Chloroform, acetonitrile, methanol, ammoinum acetate, sodium hydroxide were purchased from Merck company (Darmstadt, Germany). Double distilled water was used for the preparation of aqueous solutions.

*Instrumentation and operating conditions*

A liquid chromatographic system consisted of a 1100 series pump, a 2-channel ERC-3315 degasser, a 1200 CE detector (UV-Vis) and an interface box, all from Cecil company (Cambridge, UK); and a Nova-Pak C_18_ column with dimensions of 3.9 × 150 mm from Waters Co. (Massachusetts, US). The mobile phase consisted of 25 mM ammonium acetate (pH 7.0)/acetonitrile (10/90, v/v) with the flow rate of 1 mLmin^-1^ and the analyte was detected using a UV detector at 220 nm. The further details of the chromatographic method were provided in a recent paper (17). The applicability of the reported method was extended to analysis of methadone in serum and urine samples in this work. The pH of solutions was measured with Metrohm 654 pH meter (Herisau, Switzerland). A Hettich (EBA-20) centrifuge (Tuttlingen, Germany) and a Labtron (LS-100) vortex shaker (Tehran, Iran) were used for centrifugation and shaking the solutions, respectively.

**Table 1:** Details of patients under methadone maintenance treatment and methadone concentrations in biological samples.

No.	Age (yr)	Height (cm)	Weight (Kg)	Dose_0_ (mg)	Duration of MMT (yr)	Sampling time (hr)	Co-administered drug	EBC (mcgL^-1^)	Serum (mcgL^-1^)	Urine (mcgL^-1^)	Corrected dose (mg)
1	46	170	67	50	5	2		603	361	7411	48
2	34	175	85	100	4	1	Clonidine	615	253	1226	98
3	42	172	72	50	3	4		513	1113	3935	45
4	32	174	67	15	2	1		652	125	4425	15
5	28	190	88	50	0	3	Propranolol, Fluoxetine, Vit B_1_	615	120	4825	47
6	41	183	87	20	2	4		572	102	1029	18
7	45	175	83	100	3	4	Propranolol, Citalopram, Vit B_1_	623	586	2890	92
8	49	160	75	90	2	12	Propranolol, Alprazolam	345	671	2275	67
9	36	175	95	60	6	3		432	276	914	56
10	35	200	94	100	2	1		369	884	5602	98
11	34	174	62	12	3	1		370	95	1128	12
12	36	180	73	200	4	6		339	369	14948	172
13	49	176	97	125	5	2	Clonidine, Lorazepam	321	789	4236	119
14	42	175	63	75	0	3		594	339	7726	70
15	53	170	75	50	1	8		521	734	2982	41
16	52	173	92	125	2	8		325	1572	11982	103
17	45	176	62	80	2	18	Propranolol, Citalopram	381	147	866	51
18	47	160	61	90	2	20		305	610	1109	55
19	48	170	65	20	2	20		584	221	795	12
20	35	176	88	25	1	1	ASA, Metoprolol	560	241	1390	25
21	42	175	104	20	2	3		521	219	1796	19
22	52	175	60	60	4	1		539	203	2704	59
23	39	170	73	55	5	4		545	401	979	50
24	34	170	70	75	2	4		550	528	2712	68
25	40	180	78	70	4	14		569	394	2648	50
26	35	179	70	100	8	16		574	355	3521	67
27	31	178	90	60	0	3		421	246	3990	56
28	50	185	110	55	3	3		307	812	4567	51
29	35	170	65	80	1	4		409	381	2838	72
30	33	173	63	45	2	1		261	201	998	44
31	44	176	78	60	2	11		416	164	2920	46
32	26	183	85	90	2	3	Amitriptyline, Chlordiazepoxide	449	1346	1162	85
33	29	173	72	30	3	1	Clonazepam	330	253	1993	29
34	25	185	65	75	2	15		364	154	4323	52
35	38	170	80	100	1	2	Propranolol, Alprazolam, Vit B_1_, Fluoxetine	839	659	2723	95
36	30	178	83	100	4	8	Alprazolam, Citalopram	245	769	3955	82
37	29	183	90	85	2	12	Propranolol	308	831	2643	63
38	32	180	95	75	2	2		330	378	3065	71
39	31	165	81	75	12	3	Clonazepam, Nortriptyline	321	784	3011	70
40	54	170	84	15	3	18	Clonazpam	285	94	860	10
41	56	170	78	12	0	1	Atenolol	360	210	742	12
42	44	175	59	25	10	1		284	459	1378	25
43	49	168	68	25	3	7		318	291	1165	21
44	36	175	68	7	1	20		282	155	5541	4
45	36	174	68	75	1	5		324	782	1374	66
46	36	168	82	150	7	1		300	775	4330	148
47	37	175	69	25	0	2		322	316	1993	24
48	47	168	70	100	2	9		412	784	2431	80
49	32	180	78	50	2	5		811	286	3176	44
50	33	175	95	75	3	3		369	542	951	70
51	34	170	60	30	4	5		271	102	822	27
52	28	178	75	40	1	1		285	86	757	40
53	56	185	83	75	5	6		229	85	880	65

**Table 2:** Methadone concentrations in EBC, Plasma and urine samples of the patients, data from (36)

Case #	EBC (pgmin^-1^)	Plasma (mcgL^-1^)	Urine (mcgL^-1^)
2	4.9	-	1190
6	-	131	-
8	330	-	>2000
9	39	1100	>2000
11	40	-	-
16	5600	550	>2000
21	29800	-	>2000
34	-	55	-
35	200	6500	>2000
41	35	-	>2000
49	-	1930	>2000
51	300	640	-
52	60	3550	>2000

**Figure 1:**
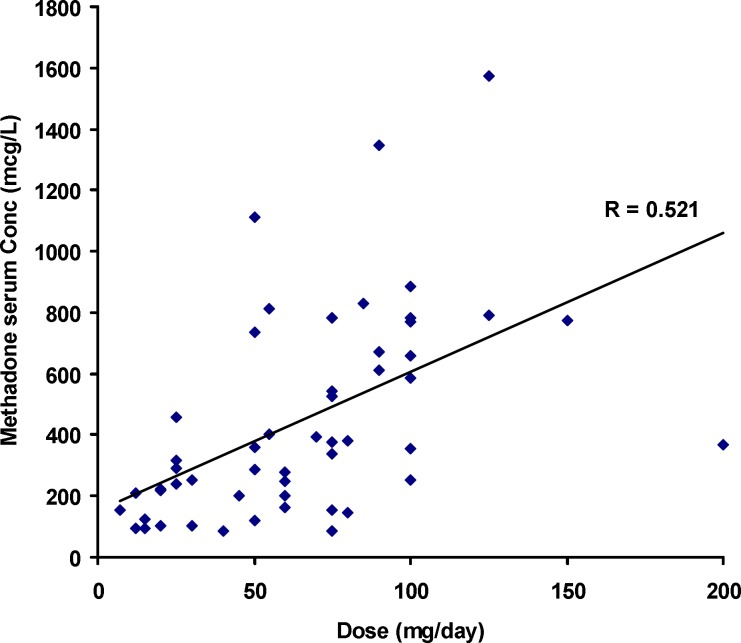
Serum concentrations of methadone versus its daily dose

**Figure 2:**
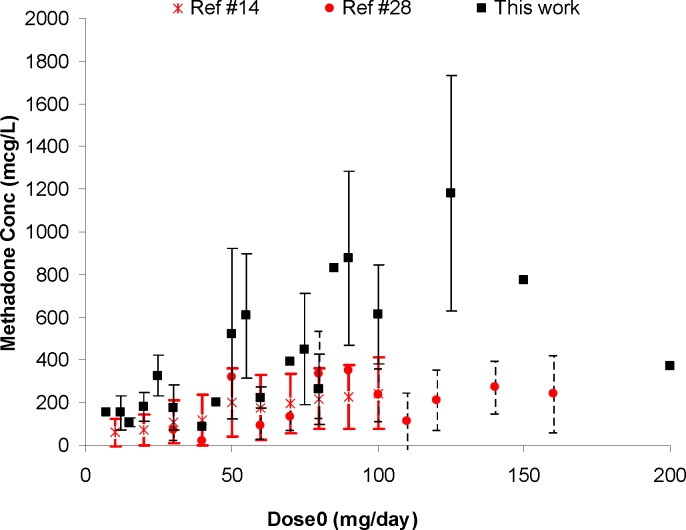
Comparison of mean   standard deviations of plasma/serum methadone concentrations against daily dose (*dose*_0_) from our work and the literature data

**Figure 3:**
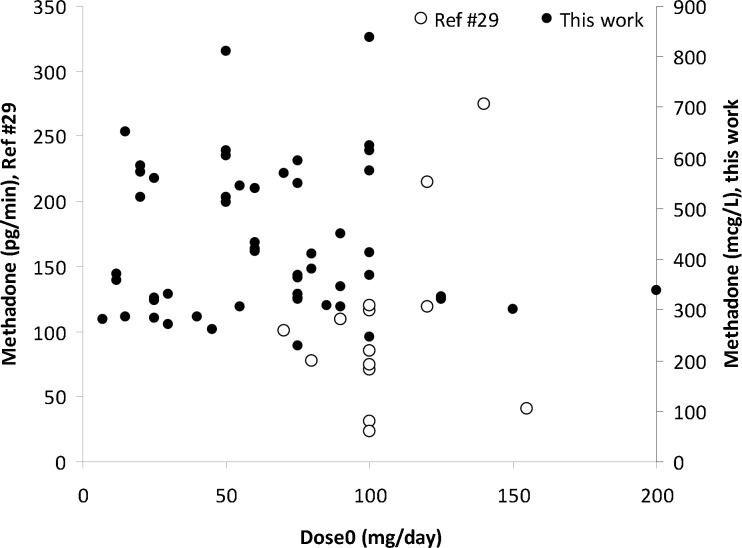
Methadone concentrations in EBC versus administered daily dose of methadone (*dose*_0_).

**Figure 4:**
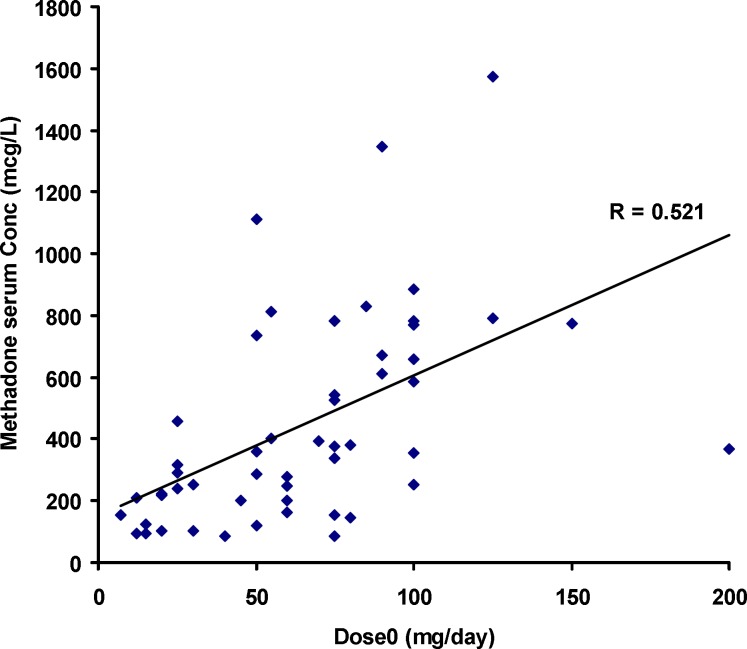
Mean and standard deviations of EBC concentrations of methadone against its daily dose (*dose*_0_

**Figure 5:**
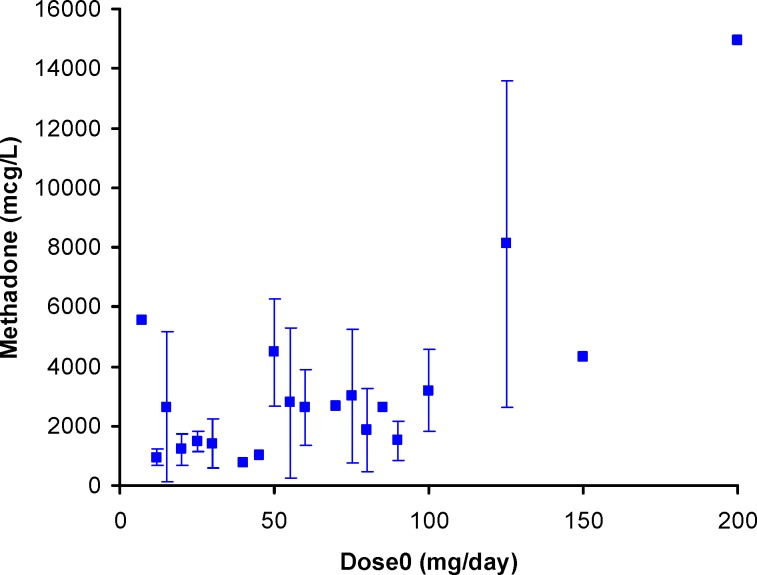
Mean and standard deviations of urine concentrations of methadone against its daily dose (*dose*_0_

*Sample preparations*

Standard stock solution of methadone (1000 mgL^−1^) was prepared in methanol and stored at 4 ºC. Working solutions were prepared by diluting with methanol. Sampling of EBC was done using a lab-made setup based on a cooling trap system patented in the national patent office (18). EBC samples were collected for 10 min and then transferred to a micro tube and stored at -80 °C until analysis. Serum and urine samples at different times after drug intake (see Table 1, column 7) were collected and centrifuged at 13000 rpm for 10 min, then stored at -80 °C until analysis. 

pH of EBC sample was adjusted (at 10.0) with sodium hydroxide (0.1 M), 1 mL of EBC was mixed with 200 µL of chloroform (as an extracting solvent), sonicated for 2 min, centrifuged for 10 min at 13000 rpm, organic phase was transferred to another micro tube, evaporated to dryness overnight, the residue was dissolved in 100 µL of the mobile phase and injected to the LC system.

1 mL of serum was mixed with 1 mL of acetonitrile (as protein precipitant and disperser solvent) in a test tube with conic bottom, centrifuged for 15 min, the supernatant transferred to another micro tube, repeatedly centrifuged for 5 min and 1 mL of the supernatant carried over to another test tube and mixed with 100 µL chloroform (as an extracting solvent), sonicated for 2 min in an ultrasonic bath, centrifuged for 10 min at 13000 rpm, and the organic phase settled to the bottom of tube transferred to a micro tube after discarding the supernatant. Finally the organic phase was evaporated to dryness overnight, the residue was dissolved in 100 µL of the mobile phase and injected to the LC system.

1 mL of urine sample was diluted (1 to 5 times) with water, pH was adjusted (to 10.0) with sodium hydroxide (0.1 M), centrifuged for 10 min at 6000 rpm, 1 mL of this sample mixed with 1 mL acetonitrile, vortexed, centrifuged for 10 min at 6000 rpm, the supernatant mixed with 200 µL of chloroform and continued with the same method as done for serum samples.

To calculate the calibration graphs in EBC, serum and urine samples, the blank samples collected from healthy volunteers were thawed at room temperature and calibration standards were prepared by spiking 1000 µL of drug-free EBC, serum and urine samples with known amounts of the drug to achieve the concentrations ranging from 0.5 to 500 µgmL^−1^ and were kept at room temperature for 20 min before use. Then the samples were treated as mentioned above and the peak heights of the samples were plotted against the added concentrations of methadone.

All sample donors have been informed on details of this study and signed a consent form approved by the Ethics Committee of Tabriz University of Medical Sciences.

*Dosage correction according to sampling time*

According to the half life definition, the dose correction after a given period of time could be done as:

$\ln\left({\mathrm{dose}}_{t}\right)=\mathrm{in}\left({\mathrm{dose}}_{0}\right)-\left(\frac{0.693}{t_{1/2}}\right)t$

where *dose*_0_ is the administered dose, *dose*_t_ is the normalized dose of methadone according to the sampling time (*t*), and *t*_½_ is the half life of methadone (*t*_½_=28 h (1)). 

## Results

All 53 patients (sampling times <24 h) were males. Mean age at admission was 39.3±8.2 years (range 25-56 years). Mean duration of methadone maintenance regime was 2.8±2.4 year (range 0-12 year). Mean weight at admission was 77.4±12.3 Kg (range 59-110 Kg). Mean height was 175.2 ± 6.9 cm (range 160-200 cm). Mean sampling time was 5.84 ± 5.86 h (range 1-20 h). The mean daily methadone dose was 65.11 ± 38.26 mg (range 7-200 mg), mean corrected dose was 55.51 ± 33.62 mg (range of 3.93-168.19 mg), mean serum methadone level was 445±333 mcgL^-1^ (range 85-1572 mcgL^-1^), mean urine methadone level was 3030 ± 2696 mcgL^-1^ (range 742-14948 mcgL^-1^) and mean EBC methadone level was 430 ± 146 mcgL^-1^ (range 229-839 mcgL^-1^). Methadone has been determined in all patient serum, urine and EBC samples. Details of data are listed in Table 1.

Figure 1 shows the individual serum concentrations of 53 patients versus daily dose of methadone. Very wide ranges of methadone serum concentrations were observed, as an example, it was varied from 252 mcgL^-1^ to 884 mcgL^-1^ after oral administration of 100 mg of methadone. From another viewpoint, serum concentration of ~ 400 mcgL^-1^ was obtained for daily intake of 25 mg to 200 mg of methadone. Similar widely scattered pattern have been reported in the literature (19-25). Figure 2 compares the mean and standard deviations of plasma/serum concentrations of methadone against daily dose of our data and those from the literature (14, 26). Wide variations are still observed for mean values from our and others works.

Scattered graph was obtained for methadone concentrations in EBC against its daily dose as shown in Figure 3. A number of reasons could be considered for these wider variations in EBC data; including variations in distribution of methadone into lung lining fluid, content of exhaled water, the humidity of the EBC sample collection area, and the individual variations which also apply for serum and urine samples. A similar pattern was reported by Beck *et al.* (27). Dose-concentration profile of methadone is still scattered when mean of concentrations were plotted against administered dose (see Figure 4). Figure 5 shows the mean ± standard deviations of urine concentrations of methadone against daily dose.

Significant correlation was observed between *dose*_t_ and serum concentrations of methadone (Pearson correlation coefficient of 0.521, N=53 and *p*<0.0005) as shown in Figure 1. The correlation coefficient was decreased to 0.436 (*p*<0.001) when was considered. 

The corresponding correlations for methadone concentrations in urine samples were 0.537 and 0.541 (both with *p*<0.0005), respectively. A weak correlation was obtained for EBC concentrations of methadone and the *dose*_t _ or $\frac{{\mathrm{dose}}_{t}}{\mathrm{Weight}}$. In some reports, the correlations between concentrations in biological fluids and oral dose of methadone were investigated in different dose classes. No improvement is observed in our study by conducting the computations in different dose classes or by excluding patients receiving co-administered drugs (listed in 8^th^ column of Table 1). By using multiple linear regression analysis the following relationship is obtained between methadone concentrations in EBC, urine, *dose*_t_ and height of patients:

$\mathrm{EBC}=0.024\mathrm{Urine}-1.82\times 10^{-3}{\mathrm{dose}}_{t}+3.027\times 10^{-3}\mathrm{Height}$

This relationship is statistically significant with the correlation coefficient of 0.919, the F value of 90, the probability of < 0.0005 and should be further investigated using larger sample size in future studies.

*Correlation of biological methadone concentrations in serum, urine and EBC samples*

A poor correlation (R=0.315, *p*<0.022) was observed between serum and urine concentrations of methadone in the investigated patients. No significant correlation was observed for EBC concentrations with those in serum or urine samples. Transfer of methadone from blood to EBC/urine depends on a number of parameters including pH of these matrices, solubility of methadone in serum, urine and EBC, protein binding of methadone (which is high and also variable between different cases) and co-administration of other drugs. The pH value of serum is quite constant at 7.4, however it is not the case for urine (28) and/or EBC (29, 30). EBC dilution could be considered as another source of wide variations of methadone concentrations in EBC. It was claimed that non-volatile analytes could be diluted up to 12000 times. Although determination of urea, total cation concentration and conductivity measurement of lyophilized EBC were developed to estimate the degree of EBC dilution, the exact dilution of EBC is still unknown (31). 

## Discussion

Quantification of methadone in all EBC samples of the patients confirms a previous proposal of Beck *et al*. (27) on the possibility of monitoring methadone levels through EBC samples. In this work, this possibility is shown using 53 patient samples and a simple preconcentration procedure coupled with a low-cost HPLC-UV method (17) which is used instead of a more sophisticated and high-cost HPLC-MS analytical method. The applicability of the analytical method for determination of methadone in serum and urine samples was also shown in this work. This means that using a single set-up, all EBC, serum and urine samples could be analyzed. It is obvious that replacement of HPLC-MS with HPLC-UV method may provide many advantages in transferring the method from a bioanalytical laboratory to a MMT clinic where the concentrations of methadone should be determined for dose adjustment according to the personalized medicine strategies. 

Our observations on dose-concentration relationships in serum and urine samples are in good agreement with previous findings. A poor correlation (R=0.2, N=32, *P*=0.048) between methadone concentration and daily dose of methadone was reported by Charlier *et al*. (32). A better correlation (R=0.43, N=93, *p*<0.0005) was observed in another investigation conducted in Spain in which the outlier points belong to the patients receiving other drugs with methadone (23). Mohamad *et al*. (5) reported a significant correlation (Pearson R=0.534) between plasma concentrations of methadone and the administered dose. Adelson *et al*. (25) observed a Pearson correlation coefficient of 0.36 between plasma levels and methadone dosage. Excellent correlation (R=0.89, N=19, *p *<0.0005) was observed for patients receiving only methadone (23). There are some sources of wide variations in methadone concentrations in biological samples; such as individual variations in absorption, distribution, metabolism, excretion, phase of MMT (i.e. induction or maintenance phase), interactions with foods or other drugs, take home or strictly supervised prescription strategies, taking extra doses of methadone purchased from illicit sources (21) or selling a part of prescribed dose in black market, and variations due to different analytical tools employed for determination of methadone in biological samples. Wide variations of methadone concentrations and its correlation with oral dose were also observed in cancer patients receiving methadone (6). Despite of these wider variations in the correlation of daily dose and serum/plasma concentrations, several research groups suggested the use of these concentrations for dose adjustment of the patients under MMT (3, 9-14).

To the best of our knowledge, there is no report on correlation of EBC and serum/urine samples in the literature employing enough sample numbers. Dealing with inter-correlation between methadone concentrations, a poor correlation (R=0.585) was observed for blood-saliva concentrations of methadone for 19 patients (34). No correlation was reported for serum-saliva concentrations on 28 patient samples in another investigation (34). Very scattered data were reported (35) for EBC, plasma and urine samples (see Table 2). These reports support our findings on the inter-correlations between EBC, serum and urine concentrations of methadone.

Despite of poor correlations for methadone concentrations in the investigated biological samples, we have quantified methadone in all patients’ samples using the developed method which is in good agreement with findings of Beck *et al*. (35) on a limited number of methadone cases with available EBC, serum and urine data for methadone. Slightly different results were observed between urine-EBC correlation in which is two cases (of total cases of 12) negative EBC results were obtained for urine positive patients (16). Biological sampling time for urine and EBC was mentioned as one of the possible reasons for the observed discrepancies (16). Our findings confirm the earlier proposal of Beck *et al.* (15) on the potential use of EBC for follow up of methadone intake in the dose adjustment and monitoring studies.

## Conclusion

Methadone is measured in all EBC, serum and urine samples using a simple and low cost liquid chromatographic – UV detection system revealing the capability of the analytical method for routine applications in clinical chemistry. Wide variations in serum and urine, and the wider variations in EBC concentrations of methadone could be justified by its variable pharmacokinetics among different patients. Despite of these variations, EBC samples could be recommended as a non-invasive alternative to blood sampling and further investigations on standardization of EBC sampling and analysis should be conducted to make it more appropriate sampling technique in clinical practice.
